# Comparative Analysis of MicroRNA and mRNA Profiles of Sperm with Different Freeze Tolerance Capacities in Boar (*Sus* *scrofa*) and Giant Panda (*Ailuropoda* *melanoleuca*)

**DOI:** 10.3390/biom9090432

**Published:** 2019-09-01

**Authors:** Ming-Xia Ran, Ying-Min Zhou, Kai Liang, Wen-Can Wang, Yan Zhang, Ming Zhang, Jian-Dong Yang, Guang-Bin Zhou, Kai Wu, Cheng-Dong Wang, Yan Huang, Bo Luo, Izhar Hyder Qazi, He-Min Zhang, Chang-Jun Zeng

**Affiliations:** 1 College of Animal Sciences and Technology, Sichuan Agricultural University, Chengdu 611130, China; 2 China Conservation and Research Center for the Giant Panda, Wolong 473000, China; 3 Department of Veterinary Anatomy & Histology, Faculty of Bio-Sciences, Shaheed Benazir Bhutto University of Veterinary and Animal Sciences, Sakrand 67210, Sindh, Pakistan

**Keywords:** sperm, cryoinjury, freeze tolerance, boar, giant panda

## Abstract

Post-thawed sperm quality parameters vary across different species after cryopreservation. To date, the molecular mechanism of sperm cryoinjury, freeze-tolerance and other influential factors are largely unknown. In this study, significantly dysregulated microRNAs (miRNAs) and mRNAs in boar and giant panda sperm with different cryo-resistance capacity were evaluated. From the result of miRNA profile of fresh and frozen-thawed giant panda sperm, a total of 899 mature, novel miRNAs were identified, and 284 miRNAs were found to be significantly dysregulated (195 up-regulated and 89 down-regulated). Combined analysis of miRNA profiling of giant panda sperm and our previously published data on boar sperm, 46, 21 and 4 differentially expressed (DE) mRNAs in boar sperm were believed to be related to apoptosis, glycolysis and oxidative phosphorylation, respectively. Meanwhile, 87, 17 and 7 DE mRNAs in giant panda were associated with apoptosis, glycolysis and oxidative phosphorylation, respectively. Gene ontology (GO) analysis of the targets of DE miRNAs showed that they were mainly distributed on membrane related pathway in giant panda sperm, while cell components and cell processes were tied to the targets of DE miRNAs in boar sperm. Finally, Kyoto Encyclopedia of Genes and Genomes (KEGG) analysis of DE mRNAs indicated that most of these DE mRNAs were distributed in membrane signal transduction-related pathways in giant panda sperm, while those in boar sperm were mainly distributed in the cytokine-cytokine receptor interaction pathway and inflammatory related pathways. In conclusion, although the different freezing extenders and programs were used, the DE miRNAs and mRNAs involved in apoptosis, energy metabolism, olfactory transduction pathway, inflammatory response and cytokine-cytokine interactions, could be the possible molecular mechanism of sperm cryoinjury and freeze tolerance.

## 1. Introduction

Cryopreservation, as an important part of artificial insemination (AI), is the most efficient method for long-term preservation of valuable genetic material from livestock, especially in endangered wild animals [1,2]. However, the process of freeze-thawing could strongly impair the fertilizing capacity and survival of sperm by reducing sperm motility and membrane integrity, increasing ROS and oxidative stress, changing membrane permeability, decreasing membrane potential, and eventually resulting in apoptosis [3,4,5]. Furthermore, the degradation effect exerted by freeze-thawing on other components crucial for sperm fertilizing ability, such as mRNAs, microRNAs (miRNAs) and long non-coding RNA (lncRNAs), could lead to the structural and functional damages in sperm, also referred to as cryoinjuries [6,7,8]. However, the molecular mechanism of sperm cryoinjury and freeze-tolerance remain largely unknown.

Sperm derived from various species respond differently to cryopreservation as demonstrated by its distinct cold shock tolerance, and resistance to freezing and osmotic pressure [9]. Cryopreservation can also affect the chromatin integrity of sperm. Chromatin in sperm is made up of DNA and nucleoproteins, which comprise of mainly protamines (P1 and P2) and 2–15% of histone H1. However, the composition (i.e., P1 only in boar, bull, and ram sperm; P1 and P2 in human, stallion, and mouse sperm) and the ratio of P1 to P2 in different species could potentially affect the resilience of the chromatin structure to freeze-thawing procedures [6]. Moreover, susceptibility to changes in cold temperature appears to be related to the ratio of polyunsaturated fatty acids (PUFA) [2]. Compared to bovine sperm, the plasma membrane of porcine sperm contains less phosphatidylcholine (lecithin) and more phosphatidylethanolamine (cephalin) and sphingomyelin. Therefore, boar sperm is more susceptible to damage during freeze-thawing. In addition, boar sperm exhibits very low content and uneven distribution of cholesterol, with more cholesterol residing in the outer layer of plasma member than in the inner layer. Since the inner monolayer is more prone to stimulation at low temperature in this composition, the consequential reorganization of the plasma member layers could affect the function of the plasma membrane [10,11]. Furthermore, cryopreservation results in a decrease in the capacity of mitochondria to produce reactive oxygen species (ROS) in boar sperm, a phenomenon that is opposite to those observed in sperm of human, bovine, and poultry, which demonstrated noticeable increase in ROS formation, lipid peroxidation, loss of motility, and death in vitro [12]. Moreover, similar to stallion sperm, cryopreservation also reduces the responsiveness of sperm to depolarization, modulators of the internal Ca^2+^ storage and progesterone in terms of the Ca^2+^ signal [13,14]. Contrary to boar sperm, giant panda sperm appears to be strongly cryo-resistant and is capable of surviving repeated cycles of freeze-thawing [15]. Spindler and his colleagues reported that the functional capacitation of plasma membrane of giant panda sperm [16] and sperm head morphometry [15] were not be affected by the rapid rates of thawing in cryopreservation protocol, neither was the decondensation of sperm, a key step of fertilization [17]. Nevertheless, the conception and reproductive success rate of AI using frozen-thawed giant panda sperm were still unsatisfactory [17].

According to our previous work, 126 differentially expressed (DE) mRNAs were annotated to the olfactory transduction pathway in giant panda sperm, however, none of these DE mRNAs were found in boar sperm [7,18]. In fact, differences in the expression level of mRNAs and miRNAs were observed in sperm of varying freeze-tolerance and motility across various species [6,9,19]. Recently, miRNAs have been reported to be involved in the regulation of spermatogenesis [20], sperm maturation [21,22] and male fertility level [23]. In addition, our previous studies have shown that miRNAs are associated with cryoinjuries in sperm [7,8]. In the present study, we adopted a high throughput sequencing approach to explore the expression profiles of miRNA in fresh and frozen-thawed giant panda sperm. Along with our previously published miRNA and mRNA profiles of boar sperm, we aimed to explore the differences in miRNA and mRNA expression pattern between boar and giant panda sperm, which is believed to be influenced by freezing procedures and high resistance to repeated freezing, respectively.

## 2. Materials and Methods

### 2.1. Ethics Statement, Sperm Collection and Cryopreservation

All experiments involved in semen collection and treatment were conducted strictly in accordance with the Regulations of the Administration of Affairs Concerning Experimental Animals (Ministry of Science and Technology, China, revised in June 2004) and were approved by the Institutional Animal Care and Use Committee in the College of Animal Science and Technology, Sichuan Agricultural University, Sichuan, China, under permit No. S20163652. Furthermore, all experimental protocols for this study were approved by the College of Animal Science and Technology, Sichuan Agricultural University.

Semen from sexually mature giant pandas with normal fertility (N = 5) were collected by electronical stimulation from China Conservation and Research Center for the Giant Panda. Parameters related to sperm quality were determined using SQA-V (MED, Israel). Semen from 5 giant pandas was pooled and then equally divided into two groups (fresh sperm and frozen-thawed sperm). The fresh semen was immediately subjected to total RNA extraction. While, frozen-thawed sperm was cryopreserved and then subjected to RNA extraction according to previous report [24]. In brief, giant panda sperm was diluted with TEST egg yolk buffer (Irvine Scientific, Santa Ana, CA, USA) to a final concentration of 5% glycerol. The mixer was loaded into 0.25 mL straws and slowly cooled to 4 °C over 4 h, then placed at 7.5 cm above liquid nitrogen (LN) for 1 min (cryopreservation rate of −40 °C /min), followed by 2.5 cm above LN for 1 min (−100 °C /min), and finally immersed in LN till use. During thawing, the straws were immersed into a 37 °C water bath for 30 s and diluted with equal volume of HF10 (Ham’s F10 medium with 5% fetal calf serum and 25mM HEPES). For boar sperm cryopreservation, after centrifugation (5 min, 1800 rpm, 17 °C), boar sperm were diluted with lactose-egg yolk (LEY) extender (40 mL 11% β-lactose, and 10 mL hen’s egg yolk) and cooled to 4 °C at a rate of approximately 0.2 °C/min. Then, the mixer was further diluted with LEY extender to yield a final concentration of 3% glycerol. Finally, the mixtures were packaged into 0.25 mL straws (FHK, Tokyo, Japan) and equilibrated in approximately 3 cm above LN vapor for 10 min, then plunged into LN till further use.

### 2.2. Total RNA Extraction, Small RNA Library Preparation and Sequencing

Prior to total RNA extraction, semen was treated with 0.5% Triton X-100 according to previous study to eliminate somatic cell contamination [25]. For each panda sample (n = 5), total RNA extraction of fresh and frozen-thawed sperm was performed with Trizol LS Reagent (Invitrogen, Carlsbad, CA). RNA concentration, purity and RNA integrity were measured using the NanoDrop 2000 Spectrophotometer (Thermo Fisher Scientific, Wilmington, DE, USA) and the RNA Nano 6000 Assay Kit of the Agilent Bioanalyzer 2100 System (Agilent Technologies, Santa Clara, CA, USA), respectively. Then, miRNA libraries were constructed using NEBNext Ultra-small RNA Sample Library Prep Kit for Illumina (NEB, Ipswich, MA, USA) according to manufacturer’s instructions. The quality and yield after sample preparation were determined with Agilent 2100 Tape Station and Qubit 2.0, and the RNA was sequenced on an Illumina Hiseq 2500 platform.

### 2.3. Quality Analysis, Mapping, miRNA Identification and Differential Expression Analysis

Clean reads were obtained by removing reads that contained adapter, poly-N, low-qualities reads and sequences < 18 nt or > 30 nt. The designated reference genome of giant panda (Ailuropoda melanoleuca) (ftp://ftp.ncbi.nlm.nih.gov/genomes/all/GCF/000/004/335/GCF_000004335.2_AilMel_1.0) was used for sequence alignment and subsequent analysis by miRDeep2 [26]. Comparison of clean reads to Silva database, GtRNAdb database, Rfam database and Repbase were performed with Bowtie [27]. Known miRNA and novel miRNA were identified by comparing sequences with known miRNAs from miRbase using miRDeep2 [26]. DESeq R package (1.10.1), based on Reads Per Kilobase Million (TPM) and Fragments Per Kilobase Million (FPKM) algorithm [28] was used for analyzing the differential expression of miRNA between fresh and frozen-thawed giant panda sperm. The standard with an adjusted *p* < 0.01 and absolute value of log2 (Fold change) >1 were classified as differentially expressed miRNA.

### 2.4. MiRNA Target Prediction, GO and KEGG Enrichment Analyses

Potential targets of miRNA were predicted by miRanda [29] and RNAhybrid [30]. Gene ontology (GO) and Kyoto Encyclopedia of Genes and Genomes (KEGG) pathway enriched in predicted target genes of the differentially expressed miRNAs were deciphered using the GOseqR package [31] and KOBAS [32] software, respectively.

### 2.5. RT-qPCR Validation

Nine randomly selected and differentially expressed miRNAs were measured by SYBR Premix Ex Taq II (TaKaRa, Japan) on a CFX96 Real-Time PCR Detection System (Bio-Rad, CA, USA). The specific primers used for quantifying the nine transcripts were listed in Table 1. In addition, the relative expression levels were normalized to the endogenous U6 and calculated using the 2^-ΔΔCt^ method [33]. Each experiment was performed in triplicate.

### 2.6. Combined Analysis of Differentially Expressed miRNA-mRNA of Boar and Giant Panda Sperm

To compare results from the transcriptomic analysis of giant panda sperm, miRNA and mRNA profiles of fresh and frozen-thawed boar sperm were downloaded from our previous works [7,18]. Systematic analysis was performed to identify differentially expressed miRNAs and mRNAs. Subsequently, sperm cryoinjury or freeze-tolerance as related to the differential expression of miRNAs and mRNAs were explored.

### 2.7. Statistical Analysis

Statistical differences were determined by independent-samples t-test using the SPSS (version 20.0). All data were shown as the means ± SEM. *p* values < 0.05 were regarded as statistically significant.

## 3. Results

### 3.1. MiRNA Profile of Fresh and Frozen-Thawed Giant Panda Sperm

A total of 16.98 million and 19.57 million raw reads were generated from fresh and frozen-thawed giant panda sperm, respectively. After removing sequences that contain adapter, ploy-N and low-quality reads, and sequences that are smaller than 18 nt or longer than 30 nt from raw data, 13.64 million and 14.47 million clean reads were obtained after the quality control step.

The length distribution of clean reads was similar in the two libraries. The 22-nt miRNAs were the most abundant (42.76% and 33.78% in fresh and frozen-thawed giant panda sperm, respectively), followed by 23- and 21-nt miRNAs.

After mapping to the designated reference genome of giant panda, a total of 899 mature, novel miRNAs corresponding to 880 miRNA precursors (pre-miRNAs) were identified in the two libraries. Furthermore, 815 and 830 miRNAs were expressed in fresh and frozen-thawed sperm, respectively. In addition, 69 and 84 miRNAs were exclusively expressed in fresh or frozen-thawed sperm, respectively. Differential expression analysis showed that 284 miRNAs were differentially expressed (DE) between fresh and frozen-thawed giant panda sperm (Table S1). Specifically, among these DE miRNAs, 195 miRNAs were up-regulated and 89 miRNAs were down-regulated.

**Figure 1:**
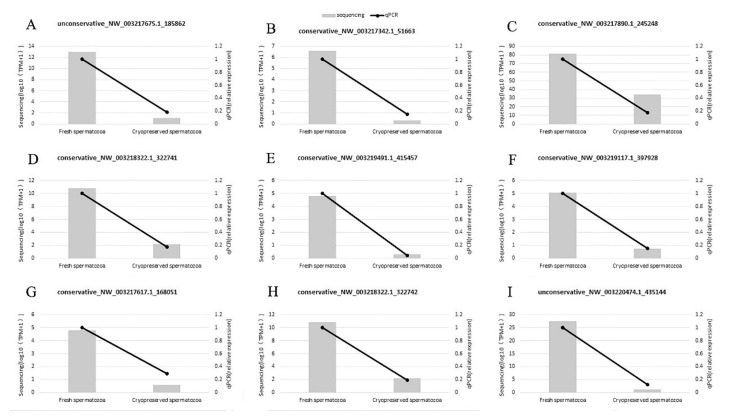
Comparison of RT-qPCR validation and RNA-sequencing results of nine randomly selected miRNAs (**A**–**I**). The column charts represent sequencing results and the line charts represent RT-qPCR results.

To validate the sequencing data, the relative expression level of nine differentially expressed miRNAs were evaluated by RT-qPCR (Figure 1). Results from RT-qPCR were consistent with RNA-seq data of all the miRNAs, which indicated that the RNA-seq results were reliable.

### 3.2. Comparison of Quality Parameters in Boar and Giant Panda Sperm Before and After Cryopreservation

Motility, viability, acrosome integrity and head morphometry of boar and giant panda sperm before and after cryopreservation were evaluated according to methods described in previous reports [15,16,18,34,35] (Table 2). The results showed that motility, viability, acrosome integrity rate and head size of giant panda sperm demonstrated less of a decrease compared to that of the boar sperm after cryopreservation. These results indicated that, based on quality parameters before and after freezing, the freeze-tolerance capacity of giant panda sperm is higher than that of boar sperm. Interestingly, head size of giant panda sperm is smaller than that of boar sperm, which is consistent with the belief that sperm with smaller heads are usually less cryopreservation-sensitive [36].

### 3.3. Combined Analysis of mRNA-miRNA Sequencing of Boar and Giant Panda Sperm

**Figure 2:**
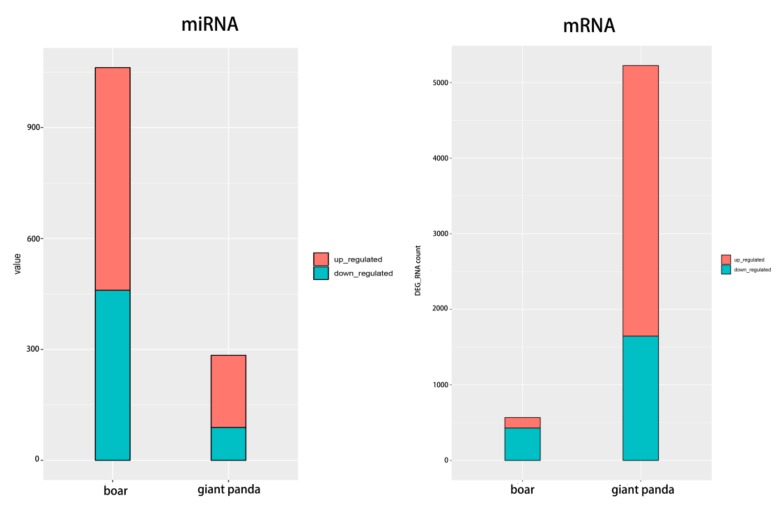
Comparison of the regulation of differentially expressed (DE) miRNAs and mRNAs in boar and giant panda sperm based on high throughput sequencing.

The number of DE mRNAs in giant panda sperm were significantly higher than that of boar sperm. On the contrary, the number of DE miRNAs in giant panda was obviously lower than that in boar sperm (Figure 2). Among the DE mRNA in boar sperm, 441 are target genes of the DE miRNAs, however, 1639 DE target mRNAs of the DE miRNAs were found in giant panda sperm (Table S2).

According to high throughput sequencing analysis, 24 DE mRNAs were identified in both boar and giant panda sperm, of which nine DE mRNAs showed the same trend of up-regulated or down-regulated expression (Table 3). Meanwhile, 15 DE mRNAs were found to demonstrate the opposite trends of expression in boar and giant panda sperm (Table 4).

### 3.4. Functional Analysis of DE mRNAs of DE miRNAs

Analysis of the target DE mRNAs of the DE miRNAs in boar sperm showed 46 apoptosis-, 21 glycolytic-, and 4 oxidative phosphorylation-related DE mRNAs. Meanwhile, 87 apoptosis-, 17 glycolytic-, and 7 oxidative phosphorylation-related DE mRNAs were found in giant panda sperm (Table S3).

### 3.5. Comparative GO and KEGG Analysis of DE miRNAs and mRNAs

**Figure 3:**
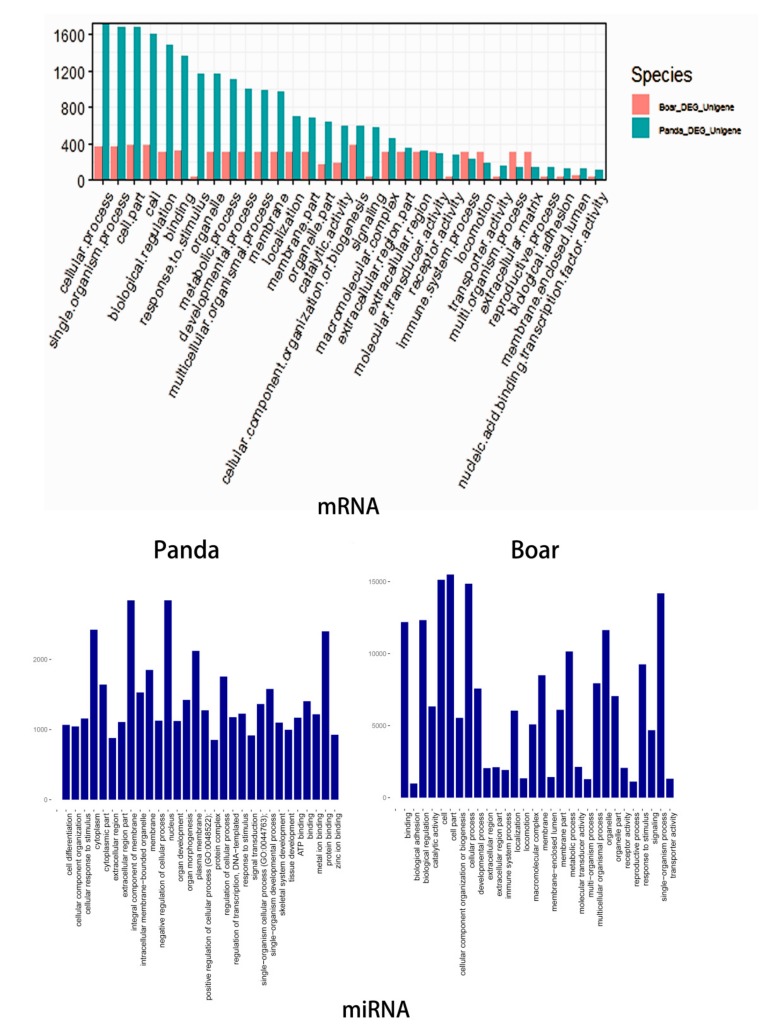
Comparative gene ontology (GO) analysis of target DE mRNAs of DE miRNAs in boar and giant panda sperm.

The DE mRNAs in boar and giant panda sperm were annotated with 61 GO terms. Of the top 30 GO terms, 28 GO terms were included in both boar and giant panda sperm. Of the 12,180 and 19,436 predicted target mRNAs, 11,684 and 61 GO terms were annotated based on the DE miRNAs in boar and giant panda sperm, respectively. The GO terms, such as binding, membrane, response to stimulus, extracellular region part, and extracellular region, were included in both boar sperm and giant panda sperm. Most of the target mRNAs of DE miRNA were largely distributed on membrane-associated GO terms in giant panda sperm. However, the target mRNAs of DE miRNA were mainly distributed on cell component and cell process terms in boar sperm (Figure 3).

**Figure 4:**
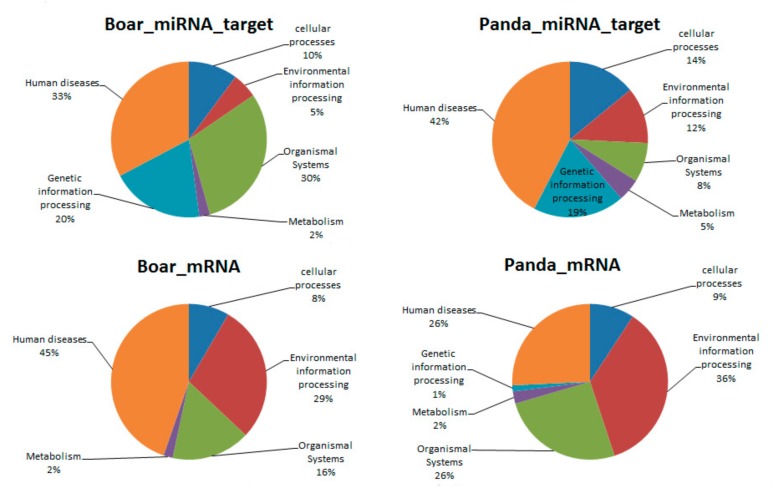
Kyoto Encyclopedia of Genes and Genomes (KEGG) pathway distribution of DE mRNAs and targets of DE miRNAs in boar and giant panda sperm.

KEGG analysis of the targets of DE miRNAs in boar and giant panda sperm showed that the differences mainly exist in environmental information processing and organismal systems (Figure 4).

**Figure 5:**
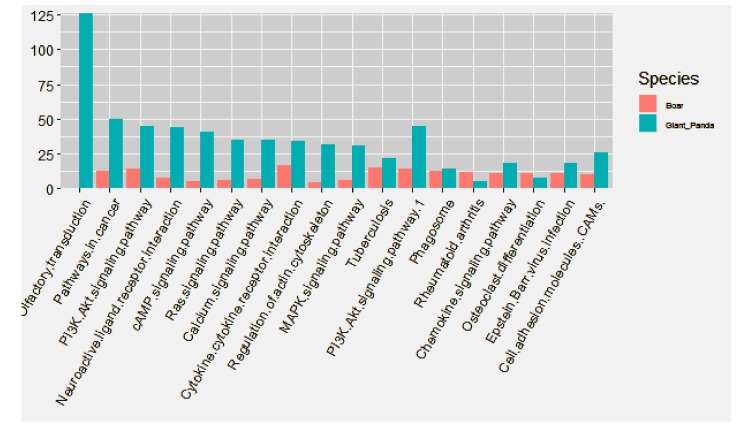
Comparison of top ten KEGG pathways of DE mRNAs in boar and giant panda sperm. The most enriched pathway was olfactory transduction, followed by pathways in cancer and PI3K-AKT signaling pathway.

Among the top 10 KEGG pathways in giant panda sperm, the DE mRNAs were mostly distributed in membrane signal transduction-related pathways, including olfactory factor transduction pathway, neuroactive ligand-receptor interaction, cAMP signaling pathway and calcium ion signaling pathway. However, the DE mRNAs in boar sperm were mainly distributed in the cytokine-cytokine receptor interaction pathway, followed by inflammatory related pathways, such as Tuberculosis pathway, Phagosome pathway and Chemokine signaling pathway (Figure 5). It is worth mentioning that no DE mRNAs were identified in the olfactory transduction pathway, which is believed to be associated with membrane depolarization, in boar sperm.

## 4. Discussion

Sperm cryopreservation is the most efficient method for long-term storage of valuable genetic material of livestock, however, the quality of post-thawed sperm varies across species. It is well known that boar sperm is susceptible to cold stimuli, attributing to the severe cryoinjuries found in boar sperm after cryopreservation. On the contrary, giant panda sperm demonstrates strong freeze tolerance and is capable of surviving repeated cycles of freeze-thawing [15]. The opposite phenomenon warrants a systematic comparison of the differences in expression of miRNAs and mRNAs in boar and giant panda sperm after cryopreservation to further understand the molecular mechanism of freeze tolerance and cryodamage. Previous studies have demonstrated an abundance of miRNAs in sperm [74], with various sperm-specific miRNA and mRNA showing various responses to cryopreservation [7,8,19,75]. The post-thaw expression of protein and RNA may be altered after cryopreservation [75,76]. Different carriers (cryostraws and cryovials) can greatly affect the proteomic profiles of human sperm during cryopreservation [77]. However, although cryopreservation seems to be harmful to human sperm DNA and compaction, the sperm DNA integrity was not affected under various cryo-storage methods [78]. According to different biochemical composition of sperm among species (e.g., ratio of polyunsaturated fatty acids), in this study, the different cryomedium and freezing programs were used to obtain the optimization effect of boar and giant panda sperm cryopreservation. Therefore, despite employing different freezing extenders and programs in this study, we speculated that this did not undermine the determination of miRNA and mRNA expression in boar and giant panda sperm.

Due to cryoinjuries, semen cryopreservation technology has yet to be widely adopted, with the pork industry being the most suspicious of this method. Studies have revealed that cryopreservation encourages apoptotic-like changes, such as mitochondrial membrane potential reduction, cysteine protease activation, membrane permeability and phosphatidylserine externalization [79] in buffalo [80], equine [81] and boar [82] sperm. The apoptosis-like changes are the main reasons for the significant decrease in sperm quality parameters, such as sperm motility, viability and acrosomal integrity [5,83,84]. Besides, the initial cooling process causes phase transitions of the membrane lipids, which further impairs the function of membrane proteins that are responsible for ion transport and metabolism [82]. Any alteration in sperm cell aptitude to utilize the substrates of adenosine triphosphate (ATP) production that provides the energy for supporting the key functions of the sperm, would expectedly compromise sperm quality and fertility. Furthermore, sperm hyperactivation and capacitation are also strongly associated with glucose metabolism [85]. Sperm ATP comes from two metabolic pathways: glycolysis and oxidative phosphorylation (OXPHOS, while sperm mitochondria produce ATP through OXPHOS, flagellum produces ATP through glycolysis [86]. Therefore, cryopreservation could affect sperm apoptosis and metabolism. In the present study, our results showed that, among the target DE mRNAs of DE miRNAs, 46 apoptosis-, 21 glycolytic-, and 4 oxidative phosphorylation related DE mRNAs were identified in boar sperm. Meanwhile, 87 apoptosis-, 17 glycolytic- and 7 oxidative phosphorylation related DE mRNAs were found in giant panda sperm. These DE mRNAs were predicted as the key genes involved in cryoinjury.

In cryopreserved bull sperm, 56 miRNAs were differentially expressed in the motile and low motile sperm populations. Furthermore, 92.8% of miRNAs were up-regulated in the motile sperm [87]. Single bull cryopreserved sperm sequencing revealed that 83 miRNAs were differentially expressed in the high and low motile sperm, and 40 miRNAs were thought to be related to apoptosis [19]. In addition, between the semen of infertile males and healthy males, 52 miRNAs were differentially expressed [88]. After thawing, a significant decrease in cholesterol content and increase in the number of acrosome-reacted sperm were observed, similar to changing during sperm capacitation [89]. Besides, our previous study revealed that miRNAs could also be involved in sperm cryoinjuries [90]. These results indicated that miRNAs could serve as critical regulators of sperm cryoinjury and cryo-resistance via inhibiting mRNA translation or degradation of mRNA.

Combined with the analysis of miRNA-mRNA interactions, we found that both the targets of DE miRNAs and the DE mRNAs in giant panda sperm were mainly distributed in the olfactory transduction pathway. Although some targets of the DE miRNAs in boar sperm were also enriched in the olfactory transduction pathway, no DE mRNAs were found to be enriched in the olfactory transduction pathway. Most of the DE mRNAs in boar sperm were enriched in the cytokine-cytokine receptor interaction pathway. We speculated that the DE mRNAs involved in the olfactory transduction pathway could be strongly associated with sperm freeze tolerance, which contributes to the higher freeze resistance capacity of giant panda sperm.

The olfactory factor transduction pathway causes an increase in intracellular calcium concentration via cAMP-activated cyclic nucleotide-gated channels (CNG), and ultimately results in membrane depolarization. In mature sperm, depolarization of the plasma membrane is essential for Ca^2+^-dependent acrosome exocytosis [13]. Ca^2+^ is a key regulator of hyperactivation of sperm motility, which is essential for fertilization [87]. The increase of intracellular calcium concentration in sperm could be related to membrane depolarization. Various Ca^2+^ channels are present on the plasma membrane of sperm, such as voltage-gated Ca^2+^ channels (Catsper), CNG, transient receptor potential channels, and sperm cation channels. During acrosome reaction, depolarization induces the opening of Catsper in plasma membrane, allowing Ca^2+^ influx and a further raise in intracellular Ca^2+^ concentration [87]. CNG channels were discovered in sea urchin sperm flagella [91]. CNG channels are physiologically active and participate in mouse sperm capacitation [92]. CNG channels are opened by the direct binding of cyclic nucleotides, cAMP and cGMP [93]. Sperm membrane-associated isoforms of adenylyl cyclases (mACs) 3 and 8 are present in both the head and the flagellum, and are regulated by several G protein-coupled receptors (GPCRs) present on sperm that produce cAMP. These GPCRs include adenosine, calcitonin, adrenergic, and odorant receptors [94]. Kobori and his colleagues found that a cyclic nucleotide-mediated process could participate in the progesterone-induced Ca^2+^ rise. Progesterone is believed to be a rapid and potent activator of human sperm fertilizing ability, and its presence could lead to a rise in cytoplasmic calcium ion concentration and plasma membrane depolarization via inducing the opening of at least two ion channels on the plasma membrane that are permeable to Ca^2+^ and Na^+^ [95]. Besides, the intracellular increase of cAMP has been suggested to play a central role in initiating the cascade of events that culminates in sperm maturation. Therefore, Ca^2+^ and Na^+^ influx induced by the opening CNG channels mediated by cAMP could play a key role in sperm plasma membrane depolarization. The depolarization of giant panda sperm membrane might be associated with cryoinjury and cryo-resistance. Besides, it has been reported that, in frozen-thawed sperm, ATP- and dbcAMP-containing extenders improved the post-thaw motility and fertilizing ability of cryopreserved rat sperm, and that cAMP concentration was lower in post-thawed sperm than in freshly ejaculated sperm [96]. Cryopreservation has also been shown to reduce the responsiveness of human and boar sperm to progesterone induced depolarization and Ca^2+^ rise [94]. These results further suggest that cAMP and Ca^2+^ could be involved in cryo-resistance of sperm. In this study, with the exception of the olfactory transduction pathway, 3 signal transduction pathways: cAMP signaling pathway, Calcium signaling pathway and Neuroactive ligand-receptor interaction, are also enriched in giant panda sperm. Furthermore, CNGB1 and CNGA3 are enriched in the olfactory transduction pathway and are significantly up-regulated in frozen-thawed giant panda sperm compared with fresh sperm. CNGA3 is predicted to be a target of three novel DE miRNAs (conservative_NW_003218630.1_359427, conservative_NW_003218630.1_359428, and unconservative_NW_003218488.1_343143). This evidence further indicates that sperm membrane signal transduction based on cAMP and Ca^2+^ may participate in quality regulation of frozen-thawed giant panda sperm.

Cytokines, which are soluble extracellular proteins or glycoproteins, are crucial intercellular regulators and mobilizers of cells. They participate in innate as well as adaptive inflammatory host defenses, cell growth, differentiation, cell death, and affect every aspect of reproductive physiology [97]. The levels of IL-6, IL-8, and TNF-α in seminal fluid, semen quality parameters, mortality and bacterial colony count are closely related to cytokine IL-18 [98]. Besides, the concentrations of IL-17 and IL-18 are negatively correlated with semen volume, density, percentage of forward motion, activity, survival rate and normal sperm morphology [99]. In the present study, some of the DE mRNAs in boar sperm are distributed in the cytokine-cytokine receptor interaction. Besides, among the top 10 pathways, DE mRNAs are also distributed in the other five inflammatory-related pathways, such as tuberculosis, phagosome and rheumatoid arthritis pathways.

## 5. Conclusions

In this study, we conducted a comparative analysis of miRNA and mRNA profiles of boar and giant panda sperm, which have been shown to have different freeze tolerance capacities. Although the different freezing extenders and programs were used, the results revealed that apoptosis, energy metabolism, olfactory transduction pathway, inflammatory response and cytokine-cytokine interactions-related DE miRNAs and mRNAs may contribute to cryoinjury and freeze tolerance. Our study provides a possible explanation for the mechanism of cryoinjury and freezing tolerance in mammalian sperm that exhibit varying cryo-resistance capacity.

## Figures and Tables

**Table 1:** Primers of differentially expressed miRNAs used in quantitative reverse transcriptase PCR (RT-qPCR).

Gene	Primer (5′–3′)
U6	F: TTATGGGTCCTAGCCTGAC
	R: CACTATTGCGGGTCTGC
unconservative_NW_003217675.1_185862	CCTGGTCGGTGATGCTCTGC
conservative_NW_003217342.1_51663	CCTGGTTCTGGAGGCTGGAAGTCTG
conservative_NW_003217890.1_245248	CTGCCCTGGCCCGAGGGACCGACT
conservative_NW_003218322.1_322741	GAGACTGTCTGGAGCCCTGGG
conservative_NW_003219491.1_415457	CCCTGGGTCGGCCGGGCTGGGGAG
conservative_NW_003219117.1_397928	CCGCCTGGACCTGGGCTACTCAA
conservative_NW_003217617.1_168051	CTGGGAGTCAGACTGTGAGG
conservative_NW_003218322.1_322742	GAGACTGTCTGGAGCCCTGGG
unconservative_NW_003220474.1_435144	TACGGGGCTGCATCAACTCTGAGGA

**Table 2:** Comparison of quality parameters of boar and giant panda sperm before and after cryopreservation.

Sperm Quality Parameters		Boar		Giant Panda	
		Fresh	Post-Thawed	Fresh	Post-Thawed
Motility (%)		92.00 ± 2.12	41.80 ± 1.78	71.70 ± 6.00	56.10 ± 3.90
Viability (%)		94.03 ± 0.68	45.19 ± 3.15	72.05 ± 6.00	63.00 ± 7.00
Acrosome integrity (%)		79.37 ± 1.43	56.26 ± 2.15	93.00 ± 1.70	81.70 ± 4.70
Sperm head	Length (µm)	8.12 ± 0.13	8.01 ± 0.12	4.7 ± 0.3	4.7 ± 0.2
	Width (µm)	4.07 ± 0.05	3.98 ± 0.04	3.6 ± 0.2	3.7 ± 0.1
	Area (μm2)	28.48 ± 0.26	27.28 ± 0.46	14.3 ± 1.4	14.7 ± 0.9
	Perimeter length (µm)	22.37 ± 0.16	21.69 ± 0.21	14.1 ± 0.8	14.2 ± 0.4

Note: Data on boar and giant panda sperm quality parameters were cited from previous reports [15,18,34,35,36].

**Table 3:** The regulation of expression and the function of the nine DE mRNAs that exhibited the same trend of expression in boar and giant panda sperm.

mRNA	Boar	Giant Panda	Functions
*GLT8D2* (Glycosyltransferase 8 Domain Containing 2)	up	up	Participates in nonalcoholic fatty liver disease (NAFLD) pathogenesis [37,38].
*CLK2* (CDC Like Kinase 2)	down	down	Acts as a hepatic gluconeogenesis and glucose output suppressor that inhibits transcriptional activity of PPARGC1A on gluconeogenic genes via its phosphorylation [39,40,41,42].
*PHACTR3* (Phosphatase and Actin Regulator 3)	up	up	Regulates cell morphogenesis, enhances cell spreading and motility through direct interaction with actin [43,44].
*RHBDD3* (Rhomboid Domain Containing 3)	up	up	Acts as a critical regulator of dendritic cell activation [45,46].
*ATAT1* (Alpha Tubulin Acetyltransferase 1)	up	up	Affects intracellular transport, cell motility, cilia formation, and neuronal signaling [47].
*METTL1* (Methyltransferase Like 1)	down	down	Mediates tRNA Methylome [48].
*DPF1* (Double PHD Fingers 1)	up	up	Promotes the formation of semen-derived virus infection enhancer and semenogelin fibrils [49].
*SERTAD4* (SERTA Domain Containing 4)	down	down	Interacts with I-mfa domain proteins and negatively regulates transcriptional activity of SERTA domain proteins [50].
*S1PR5* (Sphingosine-1-Phosphate Receptor 5)	up	up	Regulates trafficking of monocytes and influences natural killer (NK) cell distribution [51,52].

**Table 4:** The regulation of expression and the function of the DE mRNAs that exhibited the opposite trend of expression in boar and giant panda sperm.

mRNA	Boar	Giant Panda	Functions
*HSD17B14* (Hydroxysteroid 17-Beta Dehydrogenase 14)	down	up	Acts as a stereo-specific oxidation/reduction catalyst at carbon 17β of androgens and estrogens [53].
*BCAT2* (Branched Chain Amino Acid Transaminase 2)	down	up	Serves as a transporter of branched chain alpha-keto acids that catalyzes the first reaction in the catabolism of the essential branched chain amino acids leucine, isoleucine, and valine [54].
*ZNF582* (Zinc Finger Protein 582)	down	up	Involved in the aggressive progression and poor prognosis of oral squamous cell carcinoma [55].
*SLC25A25* (Solute Carrier Family 25 Member 25)	down	up	Controls adenosine triphosphate (ATP) homeostasis and contributes to the maintenance of body temperature during cold stress in mice, and is potentially involved in muscle thermogenesis under ketogenic diet (KD)-induced hypothermia in mammals [56,57].
*PRCC* (Proline Rich Mitotic Checkpoint Control Factor)	down	up	Interacts with the cell cycle control protein Mad2B, and translocates to the nucleus [58].
*MEF2D* (Myocyte Enhancer Factor 2D)	down	up	Regulates myogenesis, neuronal death and cancer cell proliferation, migration and invasion [59,60].
*ADAMTSL4* (ADAMTS Like 4)	down	up	Positively regulates apoptosis and facilitates FBN1 microfibril biogenesis [61].
*SMOX* (Spermine oxidase)	down	up	Catalyzes oxidation of spermine to generate spermidine, H_2_O_2_ and 3-aminopropanal [62].
*SLC8B1* (Solute Carrier Family 8 Member B1)	down	up	Mediates sodium-dependent calcium efflux from mitochondrion [63].
*OSM* (Oncostatin M)	down	up	Involved in inflammatory responses [64,65].
*CFP* (Complement Factor Properdin)	down	up	Acts as a positive regulator of the alternative complement pathway [66].
*RELT* (RELT TNF Receptor)	down	up	Controls the early phase of T-cell activation, probably by promoting T-cell apoptosis. Induces apoptosis in human epithelial cells [67,68].
*PELP1* (Proline, Glutamate and Leucine Rich Protein 1)	down	up	Involves in chromatin remodeling and DNA repair. PELP1-positive cells are shown to be significantly decreased in males with normal semen [69,70].
*CHST3* (carbohydrate sulfotransferase 3)	down	up	Associated with severe chondrodysplasia and progressive spinal involvement [71].
*PRAM1* (PML-RARA Regulated Adaptor Molecule 1)	down	up	Mediates retinoic acid effects in leukemia cells and stimulates the activity of HPK-1 and c-Jun N-terminal kinase (JNK) [72,73].

## Supplementary Materials

The following are available online at https://www.mdpi.com/2218-273X/9/9/432/s1, Table S1: All DE miRNAs in giant panda sperm; Table S2: DE miRNAs and their target DE mRNAs in boar and giant panda sperm; Table S3: Apoptosis, glycolysis, and oxidative phosphorylation related DE mRNAs in boar and giant panda sperm.
